# *Burkholderia gladioli* CGB10: A Novel Strain Biocontrolling the Sugarcane Smut Disease

**DOI:** 10.3390/microorganisms8121943

**Published:** 2020-12-07

**Authors:** Guobing Cui, Kai Yin, Nuoqiao Lin, Meiling Liang, Chengwei Huang, Changqing Chang, Pinggen Xi, Yi Zhen Deng

**Affiliations:** 1 Key Laboratory for Conservation and Utilization of Subtropical Agro-Bioresources, College of Plant Protection, South China Agricultural University, Guangzhou 510642, China; cuiguobing@stu.scau.edu.cn (G.C.); yinkai@stu.edu.cn (K.Y.); nancy93613@stu.scau.edu.cn (N.L.); 31000427@scau.edu.cn (M.L.);; 2 Guangdong Province Key Laboratory of Microbial Signals and Disease Control, Integrative Microbiology Research Centre, South China Agricultural University, Guangzhou 510642, China; changcq@scau.edu.cn; 3 Department of Plant Pathology, South China Agricultural University, Guangzhou 510642, China; 2019202040141@whu.edu.cn (C.H.); xpg@scau.edu.cn (P.X.)

**Keywords:** antagonistic, bio-control agent, *Burkholderia gladioli*, *Sporisorium scitamineum*, toxoflavin

## Abstract

In this study, we isolated an endophytic *Burkholderia gladioli* strain, named CGB10, from sugarcane leaves. *B. gladioli* CGB10 displayed strong inhibitory activity against filamentous growth of fungal pathogens, one of which is *Sporisorium scitamineum* that causes sugarcane smut, a major disease affecting the quality and production of sugarcane in tropical and subtropical regions. CGB10 could effectively suppress sugarcane smut under field conditions, without itself causing any obvious damage or disease, thus underscoring a great potential as a biocontrol agent (BCA) for the management of sugarcane smut. A toxoflavin biosynthesis and transport gene cluster potentially responsible for such antifungal activity was identified in the CGB10 genome. Additionally, a quorum-sensing gene cluster was identified too and compared with two close *Burkholderia* species, thus supporting an overall connection to the regulation of toxoflavin synthesis therein. Overall, this work describes the in vitro and field *Sporisorium scitamineum* biocontrol by a new B. gladioli strain, and reports genes and molecular mechanisms potentially involved.

## 1. Introduction

Plant pathogens cause significant economic losses every year, thus driving continuous research and development for novel and effective disease control strategies to ensure grain yield and food security. Sugarcane is an important economic crop in the South China area as well as other tropical or subtropical regions. Sugarcane smut disease caused by *S. scitamineum*, belonging to smut fungi, the largest group of plant pathogens, is a major constraint in sugarcane production [1]. Traditionally used fungicides face a severe challenge in maintaining the efficiency and environmental impact in controlling the major phytopathogenic fungi. Application of such fungicides has limited effect on sugarcane smut as the chemicals find it difficult to penetrate the waxy coat of the sugarcane stem, within which the dikaryotic hyphae of *S. scitamineum* thrive systemically prior to the development of visible disease lesions/symptoms [2]. Thus, biocontrol is a relatively safe alternative compared with traditional chemical control. Endophytes and/or phyllosphere-associated microbes are the main source of potential biocontrol agents [3,4]. Such endophytic bacteria generally colonize the intercellular spaces of plant tissues and can be isolated from all compartments of the plant host including the seeds [5]. These are usually symbiotic, parasitic, promotive, or pathogenic within the host plants. Endophytes have been reported as plant probiotics, with functions including nitrogen-fixation, yield increase, phytohormone production, biocontrol of plant diseases [6], and degradation of pollutants [6,7]. Increasing examples have been reported of endophytes functioning as biocontrol agents in disease management programs in crops [8,9,10].

Twenty-one genera of endophytic bacterial isolates have been reported from sugarcane tissues, with *Bacillus*, *Burkholderia*, *Enterobacter* and *Pantoea* being the dominant genera [11,12]. Several *Burkholderia* species can form either antagonistic or mutualistic interactions with fungi [13] and have also been reported as biocontrol agents, mainly based on their ability to secrete small antimicrobial compounds, including toxoflavin, chitinases, tropolone, pyrrolnitrin, etc. [13,14]. Thus far, there have been no reports about the interactions between *Burkholderia* species and sugarcane, or with a fungal pathogen(s) affecting sugarcane cultivation. In this study, we isolated and identified a novel *B. gladioli* strain, named CGB10, from sugarcane leaves. CGB10 was able to suppress the filamentous growth of several pathogenic fungi including *S. scitamineum*. CGB10 was tested as a biocontrol agent in a field trial and showed ideal effects in controlling sugarcane smut. Toxoflavin was identified as a secreted molecule from CGB10 and is likely responsible for the antifungal activity shown by CGB10. Correspondingly, toxoflavin biosynthesis and transport gene clusters were identified in the CGB10 genome. A quorum-sensing cluster was also predicted, as a potential regulator of toxoflavin production and/or transport. In summary, our study identified a potential biocontrol bacterial species for application in controlling sugarcane smut and proposed its beneficial mechanism(s) based on genomic and metabolite analyses and molecular characterization.

## 2. Methods and Materials

### 2.1. Isolation of Endophytes from Sugarcane Leaves

The sugarcane leaves used for endophyte isolation were collected from the fields of South China Agricultural University. Surface sterilization of the leaves was performed following the established protocol: the leaves were washed thrice with sterilized ultra-pure water, then soaked in 3% hydrogen peroxide for 1 min, 100% ethanol for 1 min, 6.15% hypochlorite containing Tween-20 for 5 min, 3% hydrogen peroxide for 1 min, and rinsed thoroughly with sterilized ultra-pure water at least 5–6 times. The surface-sterilized leaves were ground and mixed with 1 mL of sterilized ultra-pure water, transferred to a 2 mL sterile centrifuge tube, centrifuged at a low speed, and then the supernatant was serially diluted (10^−1^, 10^−2^, 10^−3^, 10^−4^, 10^−5^) for plating on LB medium. The LB plates were incubated at 28 °C for 3–5 days, or until bacterial colonies appeared.

### 2.2. Genome Sequencing

Genomic DNA extraction was carried out using Qiagen Genomic DNA Kit (10223). The size of DNA fragments was assessed by electrophoresis using 0.75% agarose gels. The quality of DNA was evaluated by Nanodrop (Thermo Fisher Scientific, USA), using the standard requirement of OD_260/280_ between 1.8–2.0, and OD_260/230_ between 2.0–2.2. Qubit (Invitrogen, Singapore) was used for DNA quantification. For library construction, Bluepippin automatic nucleic acid recovery instrument (Sage Science) was used for large fragment recovery, and the purified DNA fragments were end-repaired and poly-A tails added. Sequencing libraries were generated using the Genomic DNA Sequencing Kit (Oxford Nanopore Technologies, SQK-LSK108). The library was loaded onto a flow cell (FLO-MIN106) and sequenced with GridION (Oxford Nanopore Technologies).

### 2.3. Genomic Sequence Analysis

Genome structure annotation: the genome was assembled by Canu (parameter: default; version 1.7.11) and then corrected by Pilon (parameter: default; v. 1.22). Coding genes were predicted by Prodigal (parameter: pnone-g11; v. 2.6.3), and the genes with complete open reading frames were preserved; tRNA genes were predicted by tRNAscan-SE (parameter: -b-i-m lsu, SSU, TSU; v. 2.0); rRNA genes were predicted by RNAmmer (parameter: SBAC; v. 1.2); other ncRNAs were predicted by Infernal (parameter: cut_ ga -rfam --nohmonly; v. 1.1.2), and only the predictions whose lengths were more than 80% of those of their best matches in the Rfam database were preserved; CRISPRs and gene islands were predicted using Minced (parameter: default; v. 0.3.0) and Islander (parameter: default; v. 1.2), respectively.

Gene function annotation: translated protein sequences of all coding genes were analyzed by Interproscan (parameters: -appl Pfam, tigrfam, -iprloukup -goterms -t p –f tsv; v. 5.30–69.0), to identify protein domains and extract the corresponding GO annotations; BlastP (parameters: evalue 1 × 10^−5^ -outfmt ‘6 std qlen slen stitle ‘ -max_ target_seqs 5; v. 2.7.1+) was used identify best matches in KEGG and RefSeq databases (coverage greater than 30%); Rpsblast (parameter: -evalue 0.01 -SEG no -outfmt 5) was used to compare the encoded proteins to COG databases for annotation.

BLAST was used to identify gene clusters, including gbn gene cluster, toxoflavin synthesis gene cluster, and QS homologous genes. CD-HIT (parameter: default; v. 4.6.1) was used to analyze core genes (identity ≥0.4, length of query sequence ≥0.4 × length of reference sequence). The phylogenetic tree of species was constructed based on the single-copy core genes, which were converted to the protein sequences and used for multiple protein sequence alignments with MUSCLE (parameter: default, v. 3.6), followed by the phylogenetic tree construction using PhyML (maximum likelihood estimate; parament: default, v. 3.0). Average nucleotide identity (ANI) was calculated by JSpeciesWS (based on ANIm, v. 3.4.8, http://jspecies.ribohost.com/jspeciesws/).

### 2.4. Fungal Strains and Culture Conditions

Teliospores of the sugarcane smut fungus *S. scitamineum* collected from the fields in Guangdong province of China (21°12′36″ N; 101°10′12″ E) by Yan [15] were maintained at the Integrative Microbiology Research Centre (IMRC); and the *MAT-1* or *MAT-2* haploid sporidia isolated from such teliospores were used in this study. *MAT-1* (eGFP) and *MAT-2* (dsRed) generated by Yan [16] were also maintained at IMRC. The culture media used in this study includes YePSA (yeast extract 10 g/L, peptone 20 g/L, sugar 20 g/L, agar 15 g/L), PDA (Potato Dextrose Agar, MB-P1102, DingGuo, GuangZhou), and YePS liquid medium (yeast extract 10 g/L, peptone 20 g/L, sugar 20 g/L, pH = 7.0).

*P. Litchi*, *C. litchi*, *C. higginsianum*, and *F. oxysporum f.* sp. strains used in the antagonism experiments were from Z. Jiang or E. Zhou lab; *M. oryzae* was from the Deng group. The culture medium used to grow *C. litchi*, *C. higginsianum*, and *F. oxysporum f.* sp strains was PDA; for *M. oryzae* complete medium [17]; and for *P. litchi* carrot juice agar (CJA) medium [18]. Fungal mycelia were cultured at 28 °C, in constant darkness, with or without CGB10 confrontation culture, for 3–5 d before photographing.

### 2.5. Assays for Antifungal Activity

For antagonistic tests, CGB10 was grown in liquid LB medium for 12 h, and 2 μL of CGB10 culture was inoculated in the center of a PDA plate, surrounded by three pieces of fungal (*M. oryzae*, *P. Litchi*, *C. litchi*, *C. higginsianum*, and *F. oxysporum f.* sp.) mycelial plugs. For antagonism between CGB10 and *S. scitamineum*, sporidia of *MAT-1* (+) and *MAT-2* (−) mating-types were individually grown in liquid YePS medium to reach OD_600_ ≈ 1.0, then mixed with equal volume and 2 μL of such sporidial mixture was spotted onto PDA plate to surround the CGB10 inoculum. The fungal antagonism assays were performed in constant darkness at 28 °C for three days, before photographing. Three biological repeats, each containing three replicates were performed for each CGB10-fungus combination.

### 2.6. S. Scitamineum Teliospore Germination Assay

CGB10 or *E. coli* (as negative control) was pre-inoculated on a layer of cellophane on top of the PDA medium for 48 h, then the layer of bacterial inoculation was removed, leaving only the secretion from the bacteria in the growth medium, before inoculation of *S. scitamineum* teliospores on this plate. The *S. scitamineum* teliospores collected from the diseased canes were suspended in 1 mL of sterilized water, and serially diluted 10, 10^2^, 10^3^, and 10^4^ times. A 20 μL suspension of the teliospores at each dilution was streaked on the PDA plate, with or without the pre-treatment of liquid-cultured CGB10 or *E. coli*. The teliospores cultured on the PDA plates were incubated at 28 °C in constant dark conditions for 3 days for germination and promycelium formation. This experiment was repeated thrice, and each treatment contained three technical replicates. Representative images were taken with teliospores at 10^4^ dilution.

### 2.7. Detection of Toxoflavin in B. gladioli CGB10

CGB10 was cultured on PDA medium at 28 °C for 2 days, and the medium was soaked in ethyl acetate for crude extraction. The organic layer of the stratified solution was carefully transferred to the rotary evaporator for distillation until fully dry. The dried residue was dissolved in about 1 mL of methanol (grade: AR). Liquid chromatography (LC) was performed with the following settings: mobile phase: 0.1% formic acid water (*v/v*): methanol (grade: LC, methanol gradient: 0–20 min, 5–100%, 20–40 min, 100–10%); flow rate: 0.3 mL/min; retention time: about 40 min. Mass spectrometry (MS) conditions: all products were scanned by FTMS mode (Q Exactive Focus; Thermo Fisher Scientific, Germering, Germany).

### 2.8. Extracellular Hydrolytic Enzymatic Activity Test

The composition of media for semiquantitative analysis of enzymatic activities followed established protocols [19]: Pel (pectate lyase) assay medium contains 10.0 g/L of polygalacturonic acid (PGA), 10.0 g/L of yeast extract, 8.0 g/L of agarose, 0.1125 g/L of CaCl_2_, and 4.8448 g/L of Tris-HCl, pH = 8.5; Cel (cellulase) assay medium contains 1.0 g/L of carboxymethyl cellulase, 8.0 g/L of agarose, and 3.8 g/L of sodium phosphate, pH =) 7.0 [20]; Peh (polygalacturonase) assay medium contains 5.0 g/L of PGA, 2 g/L of sucrose, (NH4)_2_SO_4_ and 15 g/L of agar, pH = 5.5 [21]; and Prt (protease) assay medium contains 10.0 g/L of skimmed milk, 5.0 g/L of Bacto tryptone, 2.5 g/L of yeast extract, and 5.0 g/L of NaCl.

For preparing the enzymatic assays, 10 mL of assay medium was poured into each 10 cm circular plate, and wells of 5 mm in diameter were punched post solidification. Bacterial cells were cultured in liquid LB medium to reach OD_600_ ≈ 1.0, and 20 µL of such bacterial culture was applied to each well. The plates were incubated at 28 °C for 12 h before Pel and Peh assay plates stained with 4 M HCl, and the Cel assay plates stained with 0.1% Congo red and 1 M NaCl [21,22,23]. Halos around the wells due to protease activity became visible in Prt assay plates within 24 h without any further treatment.

### 2.9. Assessment of Bacterial Pathogenicity to Plants

Pathogenicity assays using the potato tuber slices (left panel) or onion bulbs were performed using established protocols [21,22,23,24,25]. Twenty μL bacterial culture was added to the surface of plant material and incubated at 37 °C for 24 h, before examination of disease symptoms and documentation.

Rice seed germination assay was performed as described [19]. Seeds of the rice cultivar Co-39 were soaked in 20 mL of the bacterial culture (normalized to 10^4^ CFU/mL) at room temperature for 5 h, followed by three washes with sterilized water, and transferred to moistened Whatman filter paper in a Petri dish for germination. Dishes were kept in an incubator set to 28 °C and 16 h light: 8 h dark cycle conditions. Rice seeds soaked with the same amount of sterile water served as a blank (negative) control. The experiment was repeated thrice, each of which contained 3 replicates.

### 2.10. Field Experiment for CGB10 Biocontrol of Sugarcane Smut

A disease garden was established by pouring fermented *S. scitamineum MAT-1 + MAT-2* sporidial mixture to reach an estimated inoculum of 10^6^ cells/cm^2^, in March 2019, before planting sugarcane. The smut disease appeared in the seedlings in July 2019. The sugarcane was harvested in September and allowed to re-germinate (first stubble canes) and poured with CGB10 fermentation product (2 × 10^6^ cell/cm^2^) in three randomized plots. The disease symptoms were examined in November. Each plot contained approximately 700 canes out of 200–300 clumps, and in total 2253 canes from 580 clumps, in either control plots or CGB10-treated plots. The occurrence of smut disease was calculated as a percentage of diseased canes/total canes, following an established method [26] with minor modifications.

Data Summary: The whole-genome sequence data generated and used in this study have been uploaded to the NCBI BioProject database (Accession number CP054468). Other relevant data supporting the findings of this study are available in this article and/or the Supplementary Information files.

## 3. Results

### 3.1. Genome Sequencing and Phylogenetic Analysis of CGB10

A bacterial species named CGB10 was isolated from sugarcane leaves in the farm (23.16, 113.36) at South China Agricultural University and subjected to whole-genome sequencing (For details: NCBI accession number: CP054468). The CGB10 genome was assembled into three circular sequences, including two larger chromosomes (tig00001: 4.37 Mb; and tig00002: 3.99 Mb), and a plasmid (tig00003: 165.66 Kb) (Table S1). A total of 7259 protein-coding genes were predicted (Table S2), 98% of which could be annotated by at least one of the following databases: COG, KEGG, GO, Refseq, Pfam, and TIGRFAMs (Table S3). These functional annotations and predictions of other genomic features such as non-coding RNAs (ncRNA), CRISPR (clustered regularly interspaced short palindromic repeat sequences), as well as genomic islands, are available in Dataset S1. Functional annotations with the COG database or KEGG database are illustrated in Figures S1 and S2.

**Figure 1:**
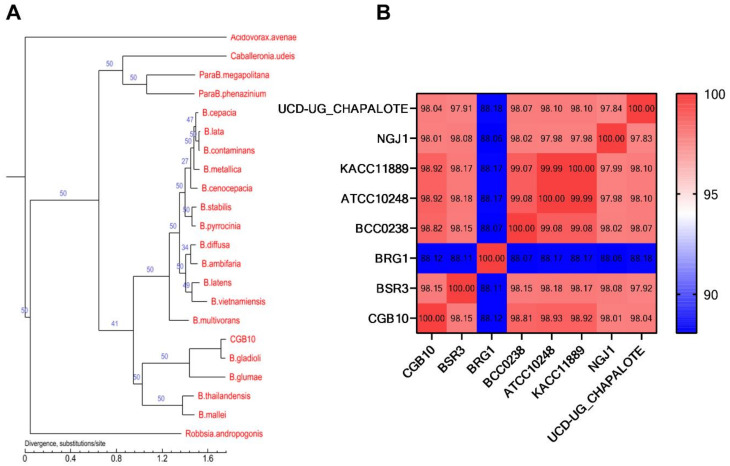
Phylogenic analysis of CGB10. (**A**) Phylogenic analysis based on single-copy core genes. The number on the branch indicates the reliability of the branch, and the closer the value to 50, the higher the reliability; the length of the branch indicates the size of the evolutionary distance, which is calculated by the average number of substitutions of each amino acid. (**B**) ANI analysis based on the whole genome of the CGB10 strain in comparison with six selected *B. gladioli* strains and a *B. glumae* strain BGR1.

We compared the CGB10 genome to 21 available genome sequences (in the order of Burkholderiales [27]; Dataset S2) for a better phylogenetic classification. In total, we identified 28,583 genes that were present in at least one of the 22 analyzed genomes, among which 763 single-copy core genes were shared by all the genomes (Figure S3; Dataset S2). We then reconstructed the phylogeny of the 22 bacterial species based on these 763 single-copy core genes, and the results showed that CGB10 was closest to *B. gladioli*, and together they clustered with *B. glumae* (Figure 1A). We further confirmed the taxonomic status of CGB10 by calculating the average nucleotide identity (ANI) [28] based on the whole genome of the CGB10 strain in comparison with another six *B. gladioli* strains, including established rice pathogen BSR3 [24], strains isolated from healthy plants and displaying antifungal activity [29,30,31], and strains isolated from a cystic fibrosis patient [32], as well as a *B. glume* strain BGR1 [24], which is also known as a rice pathogen (information of selected strains was detailed in Table 1). The identity between CGB10 and the six selected *B. gladioli* strains was all higher than 98%, yet the identity between CGB10 and the *B. glumae* strain BGR1 was 88.12%. This result confirmed that CGB10 was identified as a new *B. gladioli* strain.

### 3.2. Antifungal Activity of CGB10 under In Vitro Culture Condition

**Figure 2:**
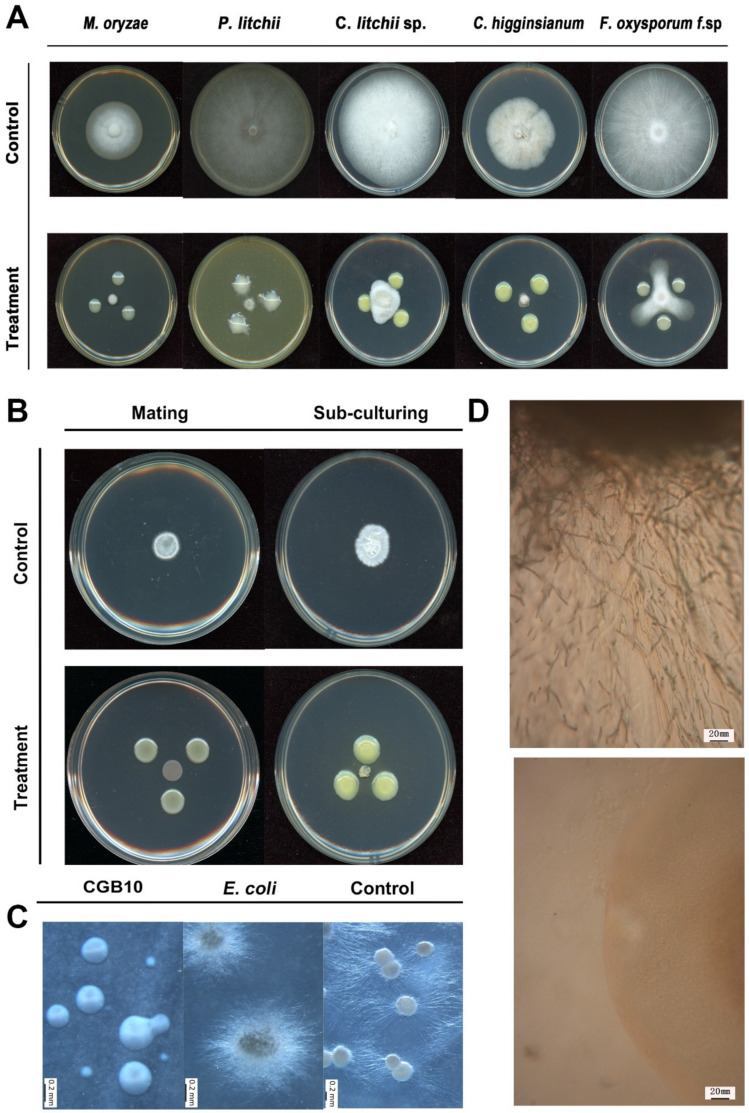
A sugarcane endophytes bacterium CGB10 could suppress the filamentous growth of multiple pathogenic fungi. (**A**) Antagonistic inoculation of CGB10 with fungal colonies on the solid medium (detailed in Materials and Methods), incubated at 28 °C for 7 d, before photographing. (**B**) Antagonistic inoculation of CGB10 with filamentous colonies of *S. scitamineum*, by mixing the sporidia of opposite mating types to induce sexual mating, or by subculturing the mycelial colony, on the solid PDA medium, incubated at 28 °C for 7 d, before photographing. (**C**) CGB10 suppressed promycelium formation from *S. scitamineum* teliospores. Liquid cultured CGB10 (OD = 1.0) was applied to the PDA plate covered with a layer of cellophane, which was removed after culturing at 28 °C for 24 h, and *S. scitamineum* teliospores were streaked on this pre-treated PDA medium to initiate germination. Scale bar = 0.2 mm. (**D**) Microscopic observation of the edge of teliospore colony with or without CGB10 pre-treatment. Scale bar = 20 mm.

Burkholderia strains include pathogens of animals, humans, and/or plants, but also members displaying beneficial properties, including antifungal activities [27]. We next tested the antifungal activity of the newly identified *B. gladioli* strain CGB10 against several well-known fungal pathogens to crops or fruits, including *Magnaporthe oryzae* (causes rice blast), *Peronophthora litchii* (litchi downy blight), *Colletotrichum siamense* (litchi pepper spot disease), *Colletotrichum higginsianum* (cauliflower anthracnose), *Fusarium oxysporum f.* sp (Panama disease of banana). CGB10 exhibited strong inhibitory activity on filamentous growth of these tested fungal pathogens, under in vitro culture conditions (Figure 2A).

We were particularly interested to see whether CGB10 was able to inhibit the filamentous/mycelial growth of the sugarcane smut fungus *S. scitamineum*, given that dikaryotic hyphae formation and growth after sexual mating is critical for *S. scitamineum* pathogenicity. We mixed an equal number of wild-type *MAT-1* (+) and *MAT-2* (−) *S. scitamineum* sporidia, and allowed them to form filaments, as showed in the untreated control (Figure 2B). In contrast, the mixed sporidial colony remained smooth as typical haploid, yeast-cell colony (Figure 2B), indicating that mating/filamentation was suppressed by CGB10. Alternatively, we subcultured the filamentous colony (wild-type *MAT-1* and *MAT-2* sporidial mixed and grown for 3 days) to a fresh medium, with or without the antagonistic CGB10 colonies. Interestingly hyphal growth of such subcultured colonies was also suppressed by CGB10 (Figure 2B). To differentiate whether CGB10′s inhibitory effect was on *S. scitamineum* sexual mating or filamentation, we used a dual-color imaging system developed by Yan [16] for visualization of dikaryon formation in the presence or absence of CGB10. When *MAT-1* (eGFP) and *MAT-2* (dsRed) fused with each other through sexual mating, the dikaryotic hyphae appeared orange or light orange under control (untreated) conditions (Figure S4). In contrast, increased haploid sporidia were observed in the presence of CGB10, indicating suppression of sexual mating. Occasionally, we observed sporidia of opposite mating types (of different fluorescent signals) fused to form hyphae, but such hyphae showed limited growth (Figure S4). We concluded that *B. gladioli* CGB10 strongly suppresses sexual mating and filamentation in *S. scitamineum*.

Next, we tested the activity of CGB10 on promycelial growth of *S. scitamineum*, during teliospore germination. Teliospore germinates and promycelium formation was normal on the blank (without pre-treatment) or negative (pre-treated with *E. coli*) control (Figure 2C). In contrast, pre-treatment with CGB10 significantly suppressed promycelium formation, resulting in smooth-edged colonies derived from teliospores (Figure 2C). Microscopic observation further confirmed that the production of promycelium was completely suppressed by CGB10 (Figure 2D).

Overall, we isolated and identified a new *B. gladioli* strain CGB10 from sugarcane leaves, which displayed broad-spectrum inhibitory activity against filamentous growth of pathogenic fungi. Particularly, CGB10 could effectively suppress dikaryotic hyphae formation in *S. scitamineum* after sexual mating, and promycelium formation during teliospore germination, both of which are important for sugarcane smut disease.

### 3.3. Antifungal Activity of CGB10 under Field Conditions

**Figure 3:**
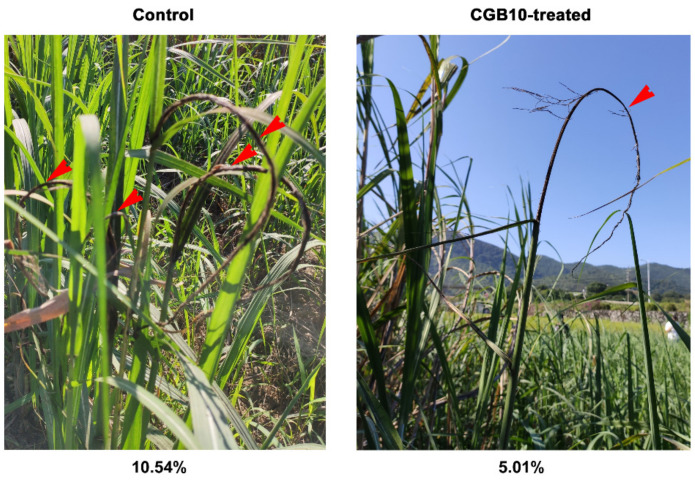
Field trial of CGB10 against sugarcane smut. Red arrows denote multiple black whips from a single clump of canes. Disease occurrence (%) was calculated based on the assessment of more than 2000 sugarcanes from approximately 500 clumps and indicated for control or CGB10-treatment respectively.

As CGB10 displayed a strong inhibitory effect on *S. scitamineum* filamentous growth after sexual mating, a critical step for its infection and pathogenesis, we considered its potential application as a biocontrol agent (BCA) in sugarcane smut. Because it was isolated from sugarcane leaves, we assumed that it may not be pathogenic/virulent to sugarcane. We next tested its antifungal activity under field conditions. More than 2000 sugarcanes from approximately 500 clumps were planted in an established disease garden of 3600 m^2^, divided into six randomized plots, three of which were with CGB10 treatment. Treatment with CGB10 did not affect the growth of sugarcane, which was expected, as it was originally isolated from sugarcane leaves. Smut disease usually occurred in multiple trunks from a single clump in the untreated control plots, while only 1–2 out of a clump in the CGB10-treated plots (Figure 3). The rate of occurrence of smut disease in CGB10-treated plots was 5.01% (diseased/total = 113/2253) and reduced by 52.46% in comparison to untreated (control) plots (10.54%; 238/2256). In the control plots, the disease canes grew short and slim, and the whips appeared from the trunks at a very early stage (Figure 3). In contrast, the canes growing in the CGB10-treated plots formed the whips at a high position, indicating a delay in symptom development upon CGB10 treatment (Figure 3). Overall, we concluded that CGB10 could effectively suppress sugarcane smut disease under field conditions.

### 3.4. Toxoflavin is the Major Filamentation-Suppressing Compound Produced by CGB10

Next, we explored the mechanism(s) underlying the antifungal activity in CGB10, from a genomic point of view. It has been reported that *B. gladioli* strains produce secondary metabolites that inhibit fungal growth [8]. Therefore, we initially searched for potential gene clusters involved in the synthesis of secondary metabolites in the CGB10 genome by using antiSAMASH (v. 5.0, https://antismash.secondarymetabolites.org). Five secondary metabolic gene clusters were predicted, including Lasalocid (t1pks), Xenoamicins, Bacteriocin, Sulfazecin, and capsular polysaccharide (Table 2). Unfortunately, our LC-MS (liquid chromatography-mass spectrometry) analysis did not detect these compounds from crude extracts of CGB10 cultured on PDA medium, suggesting that CGB10 either does not produce and/or secrete such compounds under these conditions.

It has been reported that *B. gladioli* can secrete gladiolin [33] or toxoflavin [34] as ab effective anti-microbial compound. Therefore, we set out to screen the CGB10 genome for the gene clusters involved in the synthesis of these two compounds. For gladiolin synthesis, we used gbnD1-D6, gbnF-H, and gbnI-J/gbnR-S gene clusters as reported in *B. gladioli BCC0238* strain [33,35] (Table 1) as baits. It turned out that no such gbn gene cluster is present in the CGB10 strain. On the other hand, we identified the toxoflavin biosynthesis and transporter gene clusters in the genome of CGB10, and also in all the selected *B. gladioli* and *B. glumae* strains, with Tox A-J and Tox R from *B. gladioli* BSR3 strain as baits (Table 1).

**Figure 4:**
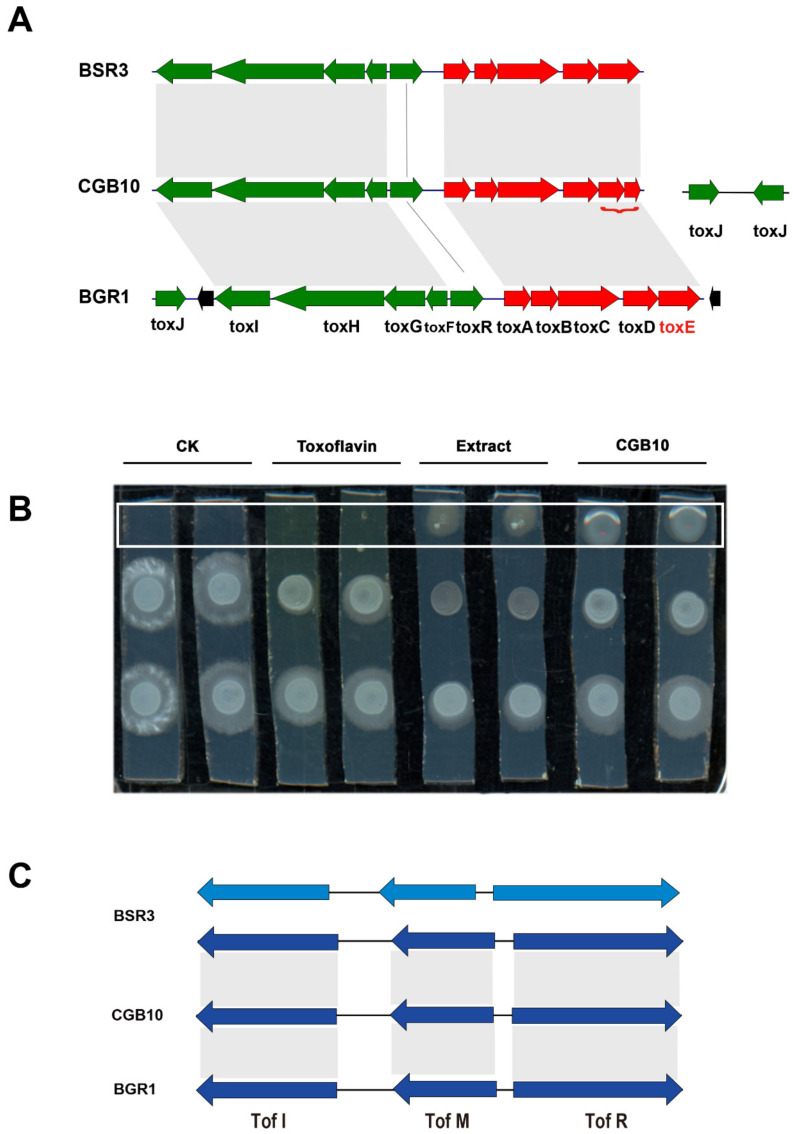
Comparison of toxoflavin biosynthesis and regulation related genes. (**A**) Comparison of toxoflavin gene cluster of CGB10, *B. glumae* BGR1, and *B. gladioli* BSR3 strains. Toxoflavin related homologous genes were mapped and aligned though local_blast (v. 2.7.1+). (**B**,**C**) Comparison of quorum-sensing gene cluster Tof I-M-R in CGB10, *B. glumae* BGR1, and *B. gladioli* BSR3 strains. Tof I-M-R homologous genes (identity >90%) were mapped though local_blast (v. 2.7.1+).

We were particularly interested in the toxoflavin gene cluster, as the CGB10 colony displayed yellow pigment (Figure 2A,B) as an indicator of toxoflavin production, and toxoflavin is known to be highly toxic to plants, fungi, animals, and microorganisms [36,37], which may contribute to CGB10′s anti-fungal-filamentous-growth activity. We further compared the toxoflavin gene cluster of CGB10 with two other reported toxoflavin-producing *Burkholderia* strains: *B. glumae* BGR1 and *B. gladioli* BSR3 [24,37]. In *B. glumae* BGR1, ToxA-E genes responsible for toxiflavin biosynthesis were under ToxR regulation, while ToxF-I genes responsible for toxiflavin transport/immunity were under ToxJ regulation. BGR1 Tox cluster genes are all located in chromosome 1 (tig1) [37]. *B. gladioli* BSR3 possesses a similar Tox cluster except that ToxJ is located on a different chromosome (tig2) [24]. CGB10′s Tox cluster is similar to BSR3, with ToxA-I on tig1, but having two ToxJ homologs (NPGAP_26430 and NPGAP_34680) on tig2 (Figure 4A, Table 3).

By LC-MS analysis we detected toxoflavin in crude extracts from CGB10 grown on PDA medium (Figure S5), confirming that CGB10 was able to produce (and secrete) toxoflavin. We also applied toxoflavin solution (Sigma-aldrich, K4394-5MG) to in vitro cultured *S. scitamineum MAT-1* and *MAT-2* mixed (1:1, *v/v*) colonies, and found a distance-dependent suppression effect on *S. scitamineum* filamentous growth, by either CGB10 crude extracts or toxoflavin (Figure 4B). Similarly, CGB10 crude extracts or pure toxoflavin displayed an inhibitory effect on *S. scitamineum* mating/filamentation (Figure S4). Overall, we proposed that toxoflavin contributes to CGB10-based suppression on mating/filamentous growth of *S. scitamineum*.

### 3.5. Identification of Quorum-Sensing Genes in CGB10

We noticed that *B. gladioli* strain KACC11889 and ATCC 10,248 were reported as not producing toxoflavin, although they possess a complete Tox gene cluster (Table 1). It has been reported that the loss of toxoflavin production in the KACC11889 strain is due to the lack of quorum-sensing (QS) system Tof I-M-R, which functions as an on/off switch for toxoflavin biosynthesis via regulation of ToxJ regulator [24,38,39]. We also searched for the Tof I-M-R cluster in the selected *B. glidioli* and *B. glumae* strains. We found that Tof I-M-R genes were absent in KACC11889 and ATCC 10,248 strains that are not producing toxoflavin, but present in all the other analyzed strains (Table 1). Although it was not reported whether toxoflavin is produced by strains NGJ1 and UCD-UG_CHAPALOTE, we inferred that they may also be capable of producing toxoflavin as they possess both the Tox gene cluster and Tof I-M-R (as a functional switch). We further compared the Tof I-M-R of CGB10 to two toxoflavin-producing *Burkholderia* strains, BSR3 and BGR1. Interestingly, we found that BSR3 contains two sets of predicted Tof I-M-R genes, located on Chromosome 2 and Plasmid 1 respectively, while BGR1 contains a set of Tof I-M-R located on chromesome2 (Table 3). CGB10 contains only one set of Tof I-M-R gene, located on tig2 (Figure 4C). Even though both CGB10 and BSR3 belong to *B. gladioli* strains, the copy number of their QS system is different, which adds more complexity to *Burkholderia* strains’ quorum sensing, regulation of toxoflavin production, and/or virulence (if any).

### 3.6. Analysis of CGB10 Pathogenicity

*B. gladioli* include many strains reported as pathogens of plants [40] or humans [41], but recent reports show that some *B. gladioli* strains are non-pathogenic or symbiotic too [42,43]. We then analyzed the CGB10 genome for potential virulence/pathogenicity factors of the following categories. First, secretory proteins, that are known as important enzymes in the life cycle of microorganisms, some of which are also important virulence factors of pathogens [42,43,44,45]. About 739 out of a total of 7259 annotated proteins of the CGB10 genome were predicted to be secreted proteins (Dataset S3), by using SignalP (http://www.cbs.dtu.dk/services/SignalP/, Hidden Markov Model). The secretion system is a channel for bacteria to secrete some proteins in order to survive, reproduce, spread, or adapt to their living environment [46,47,48]. The secretion systems in CGB10 were also predicted, including 2 T1SS, 1 T2SS, 11 T3SS, 1 T4SS, and 13 T6SS (Dataset S3). Among them, the T3SS and T6SS are known to be related to the virulence of gram-negative bacteria [48]. Correspondingly, there were 813 predicted T3SS effectors from the CGB10 genome (Dataset S3). By using the PHI database (Pathogen Host Interactions Database, v. 3.6, http://www.phi-base.org/), we obtained a total of 331 annotated proteins, 249 of which may be related to virulence based on their mutation phenotype of reduced virulence or loss of pathogenicity (Dataset S3). By BLAST in the VFPB (Virulence Factors of Pathogenic Bacteria) database (v. Tue May 5 10:06:01 2015, http://www.mgc.ac.cn/VFs/main.htm), a total of 494 virulence factors were predicted. Finally, 171 carbohydrate-active enzymes, which potentially act as bacterial virulence factors, were annotated using the CAZy database (version: 20141020, http://www.cazy.org/). These CAZy annotated proteins were further divided into six categories, namely, AA (Auxiliary Activities), CBM (Carbohydrate-Binding Module), CE (Carbohydrate Esterase), GH (Glycoside Hydrolase), GT (GlycosylTransferase), and PL (Polysaccharide Lyase), based on the activity module(s) they possess (Dataset S3). Overall, we identified potential virulence factors from the *B. gladioli* CGB10 genome by bioinformatic analyses and predicted secreted proteins or enzymes, secretion systems, and corresponding effectors, orthologs to known virulence factors too. Therefore, laboratory tests for CGB10 virulence towards plant hosts are certainly required prior to its use as a biocontrol agent.

**Figure 5:**
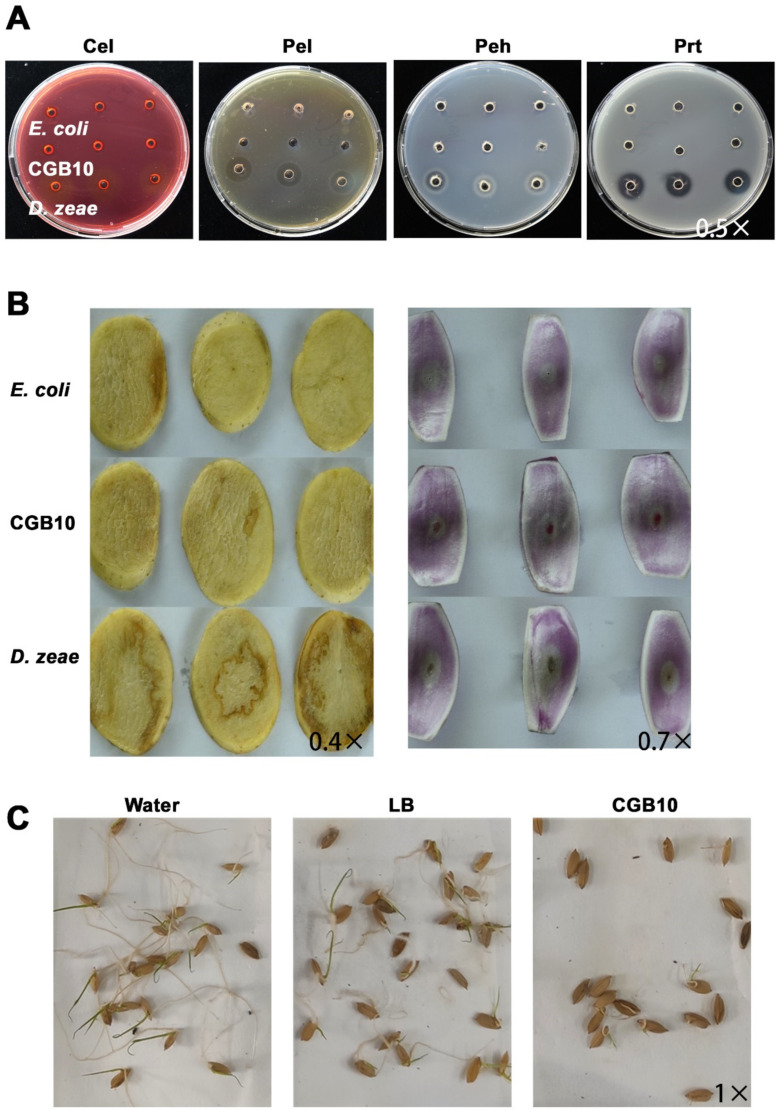
Pathogenicity assays of CGB10 on plants. (**A**) Plate assays of extracellular hydrolytic enzymatic activities. Cel (cellulase), Pel (pectate lyase), Peh (polygalacturonase), and Prt (protease) activities were examined by inoculating the bacterial cells (OD_600_ = 1.0) in the wells of plates, followed by incubation and staining for visualization of haloes around the wells as an indicator of enzymatic activity. Medium composition, incubation time and temperature, and staining protocols are detailed in Materials and Methods. (**B**) Pathogenicity assays using the potato tuber slices (left panel) or onion bulbs. From top to bottom: inoculation with *E. coli*, CGB10, and *D. zeae*, respectively. Photographs were taken at 24 h after inoculation. (**C**) Inhibitory activity of CGB10 against rice seed germination. Incubator with water or LB liquid medium served as negative controls. All the experiments were repeated three times and representative results were displayed. Magnification of each image was labeled.

We then tested the enzyme activity for Pel (pectate lyase), Peh (polygalacturonase), Cel (cellulose), and Prt (protease), which are common bacterial virulence factors in the CGB10 strain. CGB10 did not display any enzymatic activity in these aspects, as no clear zones were evident at the margin of inoculation sites (Figure 5A). *E. coli* and *Dickeya zeae* MS2 [49] were used as negative and positive controls respectively. Furthermore, we inoculated CGB10 on potato slides or on onion epidermis, to see if any disease lesion could be caused. Our results showed that CGB10 is not pathogenic to these two tested plant hosts as no damage was evident nor disease lesions formed after inoculation with CGB10 (Figure 5B). However, CGB10 was found to inhibit the germination in rice seeds (Figure 5C).

In general, we found that CGB10 did not display the same pathogenicity on onion or potato slices as reported in several other *Burkholderia* species [50], but was indeed able to suppress rice seed germination, which is similar to the rice pathogen *B. glumae* [40]. Therefore, any intention to use CGB10 as a biocontrol agent against rice fungal disease would need extra caution. Furthermore, pathogenicity assessment for CGB10 towards animal or human cells is required, to avoid potential bacteria-mediated adverse human health effects caused by CGB10.

## 4. Discussion

Bacterial species belonging to the Burkholderia genus include pathogenic and non pathogenic members [27]. Some *Burkholderia* species cause human or plant diseases, like *B. pseudomallei* and *B. mallei* are primary pathogens of animals and humans [51,52]; and *B. caryophylli* and *B. gladioli* are known as plant pathogens [51,52,53]. *B. gladioli* was also reported as a human pathogen [13,33]. On the other hand, good members of the Burkholderia genus can degrade environmental pollutions (pesticide or other contaminants) and secrete important secondary metabolites (antibiotics). *B. phenoliruptrix* sp. nov. can degrade pesticide [54] and *B. xenovorans* LB400 can degrade polychlorobiphenyls (PCBs), a type of soil pollutants [55]. *Burkholderia* sp. MSSP and *B. gladioli* possess antifungal properties, likely by secreting toxoflavin or gladiolin [33]. Therefore, it is not easy to make an immediate conclusion about whether a newly-isolated Burkholderia strain is pathogenic or beneficial.

In this study, we isolated the *B. gladioli* strain CGB10 from sugarcane leaves, and sequenced and analyzed its genome to elucidate the mechanism of its antagonistic activity against sugarcane smut fungus *S. scitamineum*. In comparison to 21 Burkholderia strains with genome sequence available on NCBI, CGB10 has 763 core genes that are common in the 22 analyzed genomes, and 520 specific genes that are only present in the CGB10 genome. Even the closest *B. gladioli* strain (ATCC 10248) has 489 specific genes that are not shared with CGB10. Therefore, it needs extra caution before we conclude whether CGB10 is a pathogen or not, although some *B. gladioli* strains were known as a plant or human pathogens.

Five secondary metabolic gene clusters were predicted in CGB10′s genome but we could not detect them by LC-MS, in this study. It could not be ruled out that the current cultivation condition may not be optimal for the biosynthesis of each of these five predicted metabolites. But under the same cultivation condition that CGB10 could effectively suppress filamentous growth of several fungal pathogens, these five secondary metabolites were not detected, indicating that they are at least not the major contributor to CGB10′s antifungal activity. On the other hand, toxoflavin may be responsible for CGB10′s antifungal activity. In comparison to two toxoflavin-producing *Buhrholderia* strain, *B. glumae* BGR1 and *B. gladioli* BSR3, the arrangement of CGB10’s toxoflavin synthesis gene cluster has some unique characteristics. *B. glumae* BGR1 possesses one-copy of regulator gene ToxJ, located approximal to the Tox F-I cluster on tig1 (chromosome 1). *B. gladioli* BSR3′s ToxJ is not located on the same chromosome (chromosome 1) as Tox A-E and Tox F-I cluster, but on another chromosome (chromosome 2) [24,56]. Instead of one, CGB10 genome has two predicted ToxJ gene, both located on a different chromosome from other Tox genes, adding complexity to the regulation of toxoflavin biosynthesis and transport in CGB10. It remains to be functionally validated which (or any) copy of CGB10 ToxJ actually regulates toxoflavin transport by inducing Tox F-I expression.

It has been reported that quorum sensing (QS) regulates toxoflavin biosynthesis in *Burkholderia* strain, as the KACC11889 strain has complete toxoflavin synthetic gene clusters but cannot secrete toxoflavin, due to its lack of QS cluster Tof I-M-R [57]. Therefore, we also compared Tof I-M-R clusters in CGB10, *B. glumae* BGR1 and *B. gladioli* BSR3. We found that the structure of Tof I-M-R cluster in CGB10 is similar to that of the known pathogen *B. gladioli* BSR3, only that BSR3 has two sets of such Tof I-M-R, while CGB10 has only one. The extra set of Tof I-M-R leads to a more complex function in rice pathogen BRS3 [58] and may account for its virulence regulation.

Potential pathogenic genes were screened from the CGB10 genome by various methods. But our pathogenicity assays using different plant materials demonstrated that CGB10 may not be virulent to plants as other established pathogenic bacteria, except that it suppressed rice seeds germination. Toxoflavin has been reported as a phytotoxin on rice seeds [24], and its antifungal or virulent effect is dose-dependent [34]. We infer that toxoflavin is also responsible for CGB10-based suppression of rice seed germination, and this reminds us to be extra cautious when applying CGB10 as a biocontrol agent against plant disease.

In the field experiment, CGB10 showed an ideal biocontrol effect on sugarcane smut. CGB10 displayed no significant suppression on sugarcane growth, likely due to the fact that it is an endophyte isolated from sugarcane. The concentration of CGB10 fermentation product to the soil is approximately 2 × 10^6^ cell/cm^2^, likely to be a concentration that is low or not toxic to a plant, animal, or human. Although *B. gladioli* strains have been well known as pathogens of plants and/or humans [40,59], recent work has demonstrated that some *B. gladioli* strains live endophytically within plants without causing any disease symptoms but displaying antifungal activity towards plant fungal pathogens [43]. We infer that such plant-endophytic *B. gladioli* strains may not be harmful to humans as they reside in the crop plants that are in close contact with agricultural practitioners. CGB10 may fall into the category of beneficial *B. gladioli* strains, as it was isolated from sugarcane leaves, and exhibits a broad-spectrum antifungal activity. Particularly, it strongly suppresses sugarcane smut caused by *S. scitamineum* under laboratory and field conditions. In conclusion, our study identified a *B. gladioli* strain that could potentially be used as a biocontrol agent against sugarcane smut, but specific evaluation for its potential virulence towards animal or human cells is surely required before application of CGB10 and/or its related products in crop protection.

## Figures and Tables

**Table 1:** Selected *B. gladioli* and *B. glumae* strains for genome comparison analysis.

Strain *	Biosample	Isolated	tofI-M-R_Chrosome(BSR3)	tofI-M-R_plasmid (BSR3)	Toxoflavin Gene Cluster (BSR3)	toxJ (BSR3)	Producing Toxoflavin	Ref.
*B. gladioli*								
KACC11889	SAMN07253176	Gladiolus	0	0	CP022005 (98.359%)	CP022006 (98.758%)	no	[24]
**BSR3**	SAMN02603164	Rice	CP002600.1 (100.000%)	CP002601.1 (100.000%)	CP002599.1 (100%)	CP002600.1 (100.000%)	yes	[24]
CGB10	SAMN15158960	Healthy sugarcane	tig00002 (98.999%)	0	tig00001 (99.065%)	tig00002 (98.646%)	yes	this study
BCC 0238	SAMEA6503627	Sputum of a child with cystic fibrosis	CADEVO010000001.1 (98.904%)	0	CADEVO010000027.1 (99.065%)	CADEVO010000022.1 (98.871%)	yes	[32]
**ATCC 10248**	SAMN03010439	*Gladiolus* sp. bulb	0	0	CP009323.1 (98.359%)	CP009322.1 (98.758%)	no	[29]
NGJ1	SAMN03764558	Healthy rice seed	LEKY01000068.1 (98.570%)	0	LEKY01000021.1 (99.000%)	LEKY01000068.1 (99.438%)	no report	[30]
UCD-UG_CHAPALOTE	SAMN03019910	Seeds of an ancient Mexican landrace of corn	JRGO01000057.1 (98.713%)	0	JRGO01000016.1 (99.355%)	JRGO01000062.1 (99.101%)	no report	[31]
*B. glumae*								
**BGR1**	SAMN02603166	Diseased rice panicle	CP001504.2 (88.353%)	0	CP001504.2 (96.703%)	CP001504.2 (82.163%)	yes	[24]

* Bold font denotes established pathogen.

**Table 2:** Prediction of secondary metabolism gene clusters.

Sequence_ID	Cluster_ID	Cluster_type	Start	End	Length	Most Similar Known Cluster	Similarity
tig00001	1	Terpene	145,180	166,019	20,840	Lasalocid (t1pks)	7%
tig00001	2	NRPS	565,719	705,139	139,421	Xenoamicins	25%
tig00001	3	NRPS, Bacteriocin	1,172,186	1,235,801	63,616	-	-
tig00001	4	NRPS	1,263,608	1,318,740	55,133	Sulfazecin	100%
tig00001	5	T1PKS	2,056,963	2,102,119	45,157	Capsular polysaccharide (Saccharide)	25%

**Table 3:** Prediction of Tox and Tof gene clusters.

Gene Name	Strain	Gene ID	Location
ToxA	BSR3	AEA59150.1	tig2
	CGB10	NPGAP_10495	tig00001
	BGR1	AAV52806.1	chromesome1
ToxB	BSR3	AEA59151.1	tig2
	CGB10	NPGAP_10490	tig00001
	BGR1	AAV52807.1	chromesome1
ToxC	BSR3	AEA59152.1	tig2
	CGB10	NPGAP_10485	tig00001
	BGR1	AAV52808.1	chromesome1
ToxD	BSR3	AEA59153.1	tig2
	CGB10	NPGAP_10480	tig00001
	BGR1	AAV52809.1	chromesome1
ToxE	BSR3	AEA59154.1	tig2
	CGB10	NPGAP_10470&NPGAP_10475	tig00001
	BGR1	AAV52810.1	chromesome1
ToxF	BSR3	AEA59148.1	tig2
	CGB10	NPGAP_10505	tig00001
	BGR1	AAV52811.1	chromesome1
ToxG	BSR3	AEA59147.1	tig2
	CGB10	NPGAP_10510	tig00001
	BGR1	AAV52812.1	chromesome1
ToxH	BSR3	AEA59146.1	tig2
	CGB10	NPGAP_10515	tig00001
	BGR1	AAV52813.1	chromesome1
ToxI	BSR3	AEA59145.1	tig2
	CGB10	NPGAP_10520	tig00001
	BGR1	AAV52814.1	chromesome1
ToxJ	BSR3	AEA63365.1	chromesome2
	CGB10	NPGAP_26430&NPGAP_34680	tig00002
	BGR1	AAV52815.1	chromesome1
ToxR	BSR3	AEA59149.1	tig2
	CGB10	NPGAP_10500	tig00001
	BGR1	AAV52816.1	chromesome1
Tof I	BSR3	bgla_2g11050	Chromesome2
	CGB10	NPGAP_35850	tig2
	BGR1	ACR31808.1	Chromesome2
Tof M	BSR3	bgla_2g11060	Chromesome2
	CGB10	NPGAP_35855	tig2
	BGR1	ACR31807.1	Chromesome2
Tof R	BSR3	bgla_2g11070	Chromesome2
	CGB10	NPGAP_35860	tig2
	BGR1	ACR31806.1	Chromesome2
Tof I-M-R (set 2)	BSR3	bgla_1p1740	plasmid1
		bgla_1p1750	
		bgla_1p1760	

## Supplementary Materials

The following are available online at https://www.mdpi.com/2076-2607/8/12/1943/s1, Figure S1: Classification of COG-annotated protein-coding sequences in the CGB10 genome, Figure S2: Classification of KEGG-annotated protein-coding sequences in the CGB10 genome, Figure S3: Pan-core genes identification in the 21 selected genomes along with CGB10, Figure S4: Expression of GFP or RFP in *S. scitamineum* sexual mating under treatment, Figure S5: LC-MS/MS identification of Toxoflavin from CGB10, Table S1: Sequence result analysis, Table S2: Statistics of genome structure prediction, Table S3: Statistics of protein-coding sequence prediction, Dataset S1: Functional annotations and predictions of other genomic features of CGB10, Dataset S2: Core-pan gene analysis, Dataset S3: Secreted proteins and pathogenicity related factors predicted in CGB10.

## References

[1] Zuo W., Okmen B., Depotter J.R.L., Ebert M.K., Redkar A., Misas Villamil J., Doehlemann G. (2019). Molecular Interactions Between Smut Fungi and Their Host Plants. Annu. Rev. Phytopathol..

[2] Sundar A.R., Barnabas E.L., Malathi P., Viswanathan R. (2014). A Mini-Review on Smut Disease of Sugarcane Caused by *Sporisorium scitamineum*. Botany.

[3] Strobel G., Daisy B., Castillo U., Harper J. (2004). Natural products from endophytic microorganisms. J. Nat. Prod..

[4] Kloeppe J.W., Rodríguez-Kábana R., Zehnder A.W., Murphy J.F., Sikora E., Fernández C. (1999). Plant root-bacterial interactions in biological control of soilborne diseases and potential extension to systemic and foliar diseases. Australas. Plant Pathol..

[5] Posada F., Vega F.E. (2005). Establishment of the fungal entomopathogen *Beauveria bassiana* (*Ascomycota: Hypocreales*) as an endophyte in cocoa seedlings (*Theobroma cacao*). Mycologia.

[6] Lodewyckx C., Vangronsveld J., Porteous F., Moore E.R.B., Taghavi S., Mezgeay M., van der Lelie D. (2002). Endophytic bacteria and their potential applications. Crit. Rev. Plant Sci..

[7] Mannisto M.K., Tiirola M.A., Puhakka J.A. (2001). Degradation of 2,3,4,6-tetrachlorophenol at low temperature and low dioxygen concentrations by phylogenetically different groundwater and bioreactor bacteria. Biodegradation.

[8] Shehata H.R., Lyons E.M., Jordan K.S., Raizada M.N. (2016). Bacterial endophytes from wild and ancient maize are able to suppress the fungal pathogen *Sclerotinia homoeocarpa*. J. Appl. Microbiol..

[9] Miller C.M., Miller R.V., Garton-Kenny D., Redgrave B., Sears J., Condron M.M., Teplow D.B., Strobel G.A. (1998). Ecomycins, unique antimycotics from *Pseudomonas viridiflava*. J. Appl. Microbiol..

[10] Cui L., Yang C., Wei L., Li T., Chen X. (2020). Isolation and identification of an endophytic bacteria *Bacillus velezensis* 8-4 exhibiting biocontrol activity against potato scab. Biol. Control.

[11] Mendes R., Pizzirani-Kleiner A.A., Araujo W.L., Raaijmakers J.M. (2007). Diversity of cultivated endophytic bacteria from sugarcane: Genetic and biochemical characterization of *Burkholderia cepacia* complex isolates. Appl. Environ. Microbiol..

[12] Lufeng L., Haichun C., Pengfei H., Yining D., Yixin W., Lilian H., Fusheng L., Yueqiu H. (2019). Isolation, Identification and Multiple Function Analyses of Sugarcane Endophytes. Chin. J. Trop. Crops.

[13] Vial L., Groleau M.C., Dekimpe V., Deziel E. (2007). *Burkholderia* diversity and versatility: An inventory of the extracellular products. J. Microbiol. Biotechnol..

[14] Schmidt S., Blom J.F., Pernthaler J., Berg G., Baldwin A., Mahenthiralingam E., Eberl L. (2009). Production of the antifungal compound pyrrolnitrin is quorum sensing-regulated in members of the *Burkholderia cepacia* complex. Environ. Microbiol..

[15] Yan M., Zhu G., Lin S., Xian X., Chang C., Xi P., Shen W., Huang W., Cai E., Jiang Z. (2016). The mating-type locus b of the sugarcane smut *Sporisorium scitamineum* is essential for mating, filamentous growth and pathogenicity. Fungal Genet. Biol..

[16] Yan M., Cai E., Zhou J., Chang C., Xi P., Shen W., Li L., Jiang Z., Deng Y.Z., Zhang L.H. (2016). A Dual-Color Imaging System for Sugarcane Smut Fungus *Sporisorium scitamineum*. Plant Dis..

[17] Deng S., Sun W., Dong L., Cui G., Deng Y.Z. (2019). MoGT2 Is Essential for Morphogenesis and Pathogenicity of *Magnaporthe oryzae*. Msphere.

[18] Jiang L., Situ J., Deng Y.Z., Wan L., Xu D., Chen Y., Xi P., Jiang Z. (2018). PlMAPK10, a Mitogen-Activated Protein Kinase (MAPK) in *Peronophythora litchii*, Is Required for Mycelial Growth, Sporulation, Laccase Activity, and Plant Infection. Front. Microbiol..

[19] Lv M., Hu M., Li P., Jiang Z., Zhang L.H., Zhou J. (2019). A two-component regulatory system VfmIH modulates multiple virulence traits in *Dickeya zeae*. Mol. Microbiol..

[20] Chatterjee A., Cui Y., Liu Y., Dumenyo C.K., Chatterjee A.K. (1995). Inactivation of rsmA leads to overproduction of extracellular pectinases, cellulases, and proteases in *Erwinia carotovora subsp*. carotovora in the absence of the starvation/cell density-sensing signal, N-(3-oxohexanoyl)-L-homoserine lactone. Appl. Environ. Microbiol..

[21] Scott-Craig J.S., Panaccione D.G., Cervone F., Walton J.D. (1990). Endopolygalacturonase is not required for pathogenicity of *Cochliobolus carbonum* on maize. Plant Cell.

[22] Chatterjee A.K., Thurn K.K., Tyrell D.J. (1985). Isolation and characterization of Tn5 insertion mutants of *Erwinia chrysanthemi* that are deficient in polygalacturonate catabolic enzymes oligogalacturonate lyase and 3-deoxy-D-glycero-2,5-hexodiulosonate dehydrogenase. J. Bacteriol..

[23] Barras F., Thurn K.K., Chatterjee A.K. (1987). Resolution of four pectate lyase structural genes of *Erwinia chrysanthemi* (EC16) and characterization of the enzymes produced in *Escherichia coli*. Mol. Gen. Genet..

[24] Lee J., Park J., Kim S., Park I., Seo Y.S. (2016). Differential regulation of toxoflavin production and its role in the enhanced virulence of *Burkholderia gladioli*. Mol. Plant Pathol..

[25] Jeong Y., Kim J., Kim S., Kang Y., Nagamatsu T., Hwang I. (2003). Toxoflavin Produced by *Burkholderia glumae* Causing Rice Grain Rot Is Responsible for Inducing Bacterial Wilt in Many Field Crops. Plant Dis..

[26] Wan-Kuan S., Zhan-Duan Y., Fu-Ye L. (2014). Identification and evaluation of some sugarcane varieties or clones for smut resistance. J. Huazhong Agric. Univ..

[27] Eberl L., Vandamme P. (2016). Members of the genus *Burkholderia*: Good and bad guys. F1000Res.

[28] Goris J., Konstantinidis K.T., Klappenbach J.A., Coenye T., Vandamme P., Tiedje J.M. (2007). DNA-DNA hybridization values and their relationship to whole-genome sequence similarities. Int. J. Syst. Evol. Microbiol..

[29] Johnson S.L., Bishop-Lilly K.A., Ladner J.T., Daligault H.E., Davenport K.W., Jaissle J., Frey K.G., Koroleva G.I., Bruce D.C., Coyne S.R. (2015). Complete genome sequences for 59 *burkholderia* isolates, both pathogenic and near neighbor. Genome Announc..

[30] Jha G., Tyagi I., Kumar R., Ghosh S. (2015). Draft Genome Sequence of Broad-Spectrum Antifungal Bacterium *Burkholderia gladioli* Strain NGJ1, Isolated from Healthy Rice Seeds. Genome Announc..

[31] Shehata H.R., Ettinger C.L., Eisen J.A., Raizada M.N. (2016). Genes Required for the Anti-fungal Activity of a Bacterial Endophyte Isolated from a Corn Landrace Grown Continuously by Subsistence Farmers Since 1000 BC. Front. Microbiol..

[32] Webster G., Jones C., Mullins A.J., Mahenthiralingam E. (2020). A rapid screening method for the detection of specialised metabolites from bacteria: Induction and suppression of metabolites from *Burkholderia* species. J. Microbiol. Methods.

[33] Song L., Jenner M., Masschelein J., Jones C., Bull M.J., Harris S.R., Hartkoorn R.C., Vocat A., Romero-Canelon I., Coupland P. (2017). Discovery and Biosynthesis of Gladiolin: A *Burkholderia gladioli* Antibiotic with Promising Activity against Mycobacterium tuberculosis. J. Am. Chem. Soc..

[34] Li X., Li Y., Wang R., Wang Q., Lu L. (2019). Toxoflavin Produced by *Burkholderia gladioli* from Lycoris aurea Is a New Broad-Spectrum Fungicide. Appl. Environ. Microbiol..

[35] Jenner M., Jian X., Dashti Y., Masschelein J., Hobson C., Roberts D.M., Jones C., Harris S., Parkhill J., Raja H.A. (2019). An unusual *Burkholderia gladioli* double chain-initiating nonribosomal peptide synthetase assembles ‘fungal’ icosalide antibiotics. Chem. Sci..

[36] Philmus B., Shaffer B.T., Kidarsa T.A., Yan Q., Raaijmakers J.M., Begley T.P., Loper J.E. (2015). Investigations into the Biosynthesis, Regulation, and Self-Resistance of Toxoflavin in *Pseudomonas protegens* Pf-5. Chembiochem.

[37] Kim J., Kim J.G., Kang Y., Jang J.Y., Jog G.J., Lim J.Y., Kim S., Suga H., Nagamatsu T., Hwang I. (2004). Quorum sensing and the LysR-type transcriptional activator ToxR regulate toxoflavin biosynthesis and transport in *Burkholderia glumae*. Mol. Microbiol..

[38] Hussain A., Shahbaz M., Tariq M., Ibrahim M., Hong X., Naeem F., Khalid Z., Raza H.M.Z., Bo Z., Bin L. (2020). Genome re-seqeunce and analysis of *Burkholderia glumae* strain AU6208 and evidence of toxoflavin: A potential bacterial toxin. Comput. Biol. Chem..

[39] Chen R., Barphagha I.K., Karki H.S., Ham J.H. (2012). Dissection of quorum-sensing genes in *Burkholderia glumae* reveals non-canonical regulation and the new regulatory gene tofM for toxoflavin production. PLoS ONE.

[40] Naughton L.M., An S.Q., Hwang I., Chou S.H., He Y.Q., Tang J.L., Ryan R.P., Dow J.M. (2016). Functional and genomic insights into the pathogenesis of *Burkholderia* species to rice. Environ. Microbiol..

[41] Zhou F., Ning H., Chen F., Wu W., Chen A., Zhang J. (2015). *Burkholderia gladioli* infection isolated from the blood cultures of newborns in the neonatal intensive care unit. Eur. J. Clin. Microbiol. Infect. Dis..

[42] Johnston-Monje D., Raizada M.N. (2011). Conservation and diversity of seed associated endophytes in Zea across boundaries of evolution, ethnography and ecology. PLoS ONE.

[43] Ettinger C.L., Shehata H.R., Johnston-Monje D., Raizada M.N., Eisen J.A. (2015). Draft Genome Sequence of *Burkholderia gladioli* Strain UCD-UG_CHAPALOTE (Phylum Proteobacteria). Genome Announc..

[44] Tjalsma H., Antelmann H., Jongbloed J.D., Braun P.G., Darmon E., Dorenbos R., Dubois J.Y., Westers H., Zanen G., Quax W.J. (2004). Proteomics of protein secretion by *Bacillus subtilis*: Separating the “secrets” of the secretome. Microbiol. Mol. Biol. Rev..

[45] Mehat J.W., Park S.F., van Vliet A.H.M., La Ragione R.M. (2018). CapC, a Novel Autotransporter and Virulence Factor of *Campylobacter jejuni*. Appl. Environ. Microbiol..

[46] Coulthurst S. (2019). The Type VI secretion system: A versatile bacterial weapon. Microbiology.

[47] Cianciotto N.P., White R.C. (2017). Expanding Role of Type II Secretion in Bacterial Pathogenesis and Beyond. Infect. Immun..

[48] Bai F., Li Z., Umezawa A., Terada N., Jin S. (2018). Bacterial type III secretion system as a protein delivery tool for a broad range of biomedical applications. Biotechnol. Adv..

[49] Feng L., Schaefer A.L., Hu M., Chen R., Greenberg E.P., Zhou J. (2019). Virulence Factor Identification in the Banana Pathogen *Dickeya zeae* MS2. Appl. Environ. Microbiol..

[50] Jacobs J.L., Fasi A.C., Ramette A., Smith J.J., Hammerschmidt R., Sundin G.W. (2008). Identification and onion pathogenicity of *Burkholderia* cepacia complex isolates from the onion rhizosphere and onion field soil. Appl. Environ. Microbiol..

[51] Titball R.W., Burtnick M.N., Bancroft G.J., Brett P. (2017). *Burkholderia pseudomallei* and *Burkholderia mallei vaccines*: Are we close to clinical trials?. Vaccine.

[52] Hemarajata P., Baghdadi J.D., Hoffman R., Humphries R.M. (2016). *Burkholderia pseudomallei*: Challenges for the Clinical Microbiology Laboratory. J. Clin. Microbiol..

[53] Burkholder W.H. (1950). Sour skin, a bacterial rot of onion bulbs. Phytopathology.

[54] Coenye T., Henry D., Speert D.P., Vandamme P. (2004). *Burkholderia phenoliruptrix* sp. nov., to accommodate the 2,4,5-trichlorophenoxyacetic acid and halophenol-degrading strain AC1100. Syst. Appl. Microbiol..

[55] Martinez P., Agullo L., Hernandez M., Seeger M. (2007). Chlorobenzoate inhibits growth and induces stress proteins in the PCB-degrading bacterium *Burkholderia xenovorans* LB400. Arch. Microbiol..

[56] Kim S., Park J., Kim J.H., Lee J., Bang B., Hwang I., Seo Y.S. (2013). RNAseq-based Transcriptome Analysis of *Burkholderia glumae* Quorum Sensing. Plant. Pathol. J..

[57] Elshafie H.S., Devescovi G., Venturi V., Camele I., Bufo S.A. (2019). Study of the Regulatory Role of N-Acyl Homoserine Lactones Mediated Quorum Sensing in the Biological Activity of *Burkholderia gladioli* pv. agaricicola Causing Soft Rot of *Agaricus* spp.. Front. Microbiol..

[58] Seo Y.S., Lim J., Choi B.S., Kim H., Goo E., Lee B., Lim J.S., Choi I.Y., Moon J.S., Kim J. (2011). Complete genome sequence of *Burkholderia gladioli* BSR3. J. Bacteriol..

[59] Imataki O., Kita N., Nakayama-Imaohji H., Kida J.I., Kuwahara T., Uemura M. (2014). Bronchiolitis and bacteraemia caused by *Burkholderia gladioli* in a non-lung transplantation patient. New Microbes New Infect..

